# Inhibition of SIK2 and SIK3 induces adaptive ER-phagy and creates a therapeutic vulnerability in ovarian cancer

**DOI:** 10.21203/rs.3.rs-9933879/v1

**Published:** 2026-07-01

**Authors:** Zhen Lu, Rumeysa Ozyurt, Gamze Bildik, Weiqun Mao, Hailing Yang, Robert Bast

**Affiliations:** UT MD Anderson Cancer Center; The University of Texas MD Anderson Cancer Center; University of Texas MD Anderson Cancer Center; UT MD Anderson Cancer Center; The University of Texas, M.D. Anderson Cancer Center

**Keywords:** ER-phagy, Unfolded protein response, SIK2/3, Proteotoxic stress, Autophagic flux, PERK–ATF4 signaling

## Abstract

Cancer cells depend on protein quality control pathways to survive intrinsic and microenvironmental stress. Endoplasmic reticulum (ER)-selective autophagy (ER-phagy) maintains ER homeostasis by eliminating damaged ER and misfolded protein aggregates during ER stress. How ER stress-induced ER-phagy is regulated in cancer remains poorly understood. Salt-inducible kinases SIK2 and SIK3 (SIK2/3) are serine/threonine kinases implicated in metabolic regulation and cancer cell survival, but their roles in ER stress signaling and ER-phagy have not previously been studied.

Here, we show that genetic or pharmacologic inhibition of SIK2/3 induces proteotoxic stress and activates the unfolded protein response through the PERK and IRE1 pathways, with predominant engagement of PERK and its downstream effector ATF4. SIK2/3 inhibition promotes ER-phagy by upregulating the ER-phagy receptor CCPG1 in an ATF4-dependent manner and increasing autophagic flux, thereby enabling cancer cell survival under stress. Disruption of this adaptive response results in the accumulation of polyubiquitinated protein aggregates, induction of CHOP, and apoptotic cell death in ovarian cancer cells.

Importantly, combined treatment with the dual SIK2/3 inhibitor GRN-300 and the autophagy inhibitor chloroquine synergistically enhanced proteotoxic stress, reduced cell viability (combination index < 0.9), and triggered CHOP-dependent apoptosis. In ovarian cancer xenograft models, GRN-300 plus chloroquine markedly suppressed tumor growth and significantly prolonged survival compared with either monotherapy. Together, these findings identify SIK2/3 as key regulators of ER stress-induced ER-phagy and reveal a targetable stress-adaptation pathway that can be exploited therapeutically in ovarian cancer.

## Introduction

Cancer cells depend on protein quality control pathways to survive intrinsic stress imposed by rapid proliferation and extrinsic stress from the tumor microenvironment, including hypoxia, nutrient limitation, and therapeutic insult (1,2). Central to this adaptive capacity is the endoplasmic reticulum (ER), which governs protein folding, lipid biosynthesis, and calcium homeostasis. Disruption of ER function results in accumulation of misfolded proteins and activation of ER stress responses that, if unresolved, compromise cellular viability (3,4).

To restore ER homeostasis, cells engage the unfolded protein response (UPR), a conserved signaling network composed of three principal branches: PERK, IRE1, and ATF6 (5–8). UPR activation transiently promotes survival by enhancing protein folding capacity, limiting global protein synthesis, and facilitating clearance of misfolded proteins. However, when ER stress is excessive or prolonged, adaptive signaling gives way to apoptotic programs, largely mediated by the transcription factor CHOP (9,10). High-grade serous ovarian cancers are particularly reliant on UPR signaling due to elevated basal proteotoxic stress driven by genomic instability, rapid proliferation, and hostile tumor microenvironments (11–13). Cytotoxic chemotherapy further exacerbates ER stress, underscoring the importance of adaptive ER stress responses in ovarian cancer progression and therapy resistance (14).

Selective autophagy pathways play a critical role in resolving ER stress by removing damaged ER membranes and aggregated proteins. ER-selective autophagy (ER-phagy) enables cells to maintain ER integrity and proteostasis under stress conditions, thereby supporting survival (15–17). ER-phagy is mediated by ER-resident receptors such as FAM134B, SEC62, and CCPG1, which link damaged ER regions to the autophagic machinery (18–20). Although ER-phagy has emerged as an important adaptive mechanism in stressed cancer cells (21,22), the upstream regulatory pathways that couple oncogenic or stress-responsive signaling to ER-phagy activation remain incompletely understood.

Salt-inducible kinases SIK2 and SIK3 (SIK2/3) are serine/threonine kinases implicated in metabolic regulation, stress adaptation, and cancer cell survival (23–25). SIK2 has been linked to ovarian cancer progression and ER-associated degradation, while the role of SIK3 in cellular stress responses remains less defined (26–29). Whether SIK2/3 coordinates ER stress signaling and adaptive ER-phagy programs in cancer cells have not been explored. GRN-300, a selective dual inhibitor of SIK2/3, provides an opportunity to interrogate these pathways and evaluate their functional significance in ovarian cancer.

In this study, we demonstrate that inhibition of SIK2/3 induces ER stress and activates a PERK-ATF4-dependent ER-phagy program mediated by the ER-phagy receptor CCPG1. We show that this selective autophagy response functions as an adaptive survival mechanism that restrains proteotoxic stress. Importantly, blockade of autophagic flux converts SIK2/3 inhibition-induced ER stress into CHOP-dependent apoptotic cell death, revealing a therapeutically exploitable vulnerability. Our findings identify SIK2/3 as key regulators of ER stress adaptation and support combining targeting of SIK2/3 and autophagy as a strategy to enhance cytotoxicity in ovarian cancer.

## Materials and Methods

### Cell lines

The cell lines utilized in this study are detailed in Supplemental Table 1. Their identities were verified through short tandem repeat (STR) DNA profiling conducted by the Characterized Cell Line Core Facility at MD Anderson Cancer Center. All cell cultures were maintained at 37°C in a humidified incubator containing 5% CO_2_. Routine screening for mycoplasma contamination was performed using the Universal Mycoplasma Detection Kit (ATCC, catalog no. 30–1010K).

### Antibodies

All antibodies were obtained commercially: Antibodies against SIK2 (Cell Signaling Technology Cat# 6919, RRID:AB_10830063), SIK3 (Cell Signaling Technology Cat# 39477, RRID:AB_3251492), BIP (Cell Signaling Technology Cat# 3177, RRID:AB_2119845), PERK (Cell Signaling Technology Cat# 3192, RRID:AB_2095847), ATF6 (Cell Signaling Technology Cat# 65880, RRID:AB_2799696), IRE1α/ERN1 (Cell Signaling Technology Cat# 3294, RRID:AB_823545), Phospho-eIF2alpha (Cell Signaling Technology Cat# 3398, RRID:AB_2096481), ATF-4 (Cell Signaling Technology Cat# 11815, RRID:AB_2616025), DDIT3/CHOP (Cell Signaling Technology Cat# 2895, RRID:AB_2089254), CASP3/Cleaved Caspase-3 (Cell Signaling Technology Cat# 9661, RRID:AB_2341188), Ubiquitin (Cell Signaling Technology Cat# 58395, RRID:AB_3075532), CCPG1 (#80158T), LC3B (Cell Signaling Technology Cat# 2775, RRID:AB_915950), and GAPDH (Cell Signaling Technology Cat# 2118, RRID:AB_561053) were purchased from Cell Signaling Technology. Anti-VCL/vinculin (Sigma-Aldrich Cat# V9264, RRID:AB_10603627) was from Sigma. The antibody against Phospho-PERK/EIF2AK3 (Thermo Fisher Scientific Cat# MA5–15033, RRID:AB_10980432) was purchased from Invitrogen.

### Immunoblotting

For protein extraction, cells were washed twice with ice-cold PBS (Corning, 21–040-CV) and lysed directly in wells with 150 μl SDS sample buffer containing 62.5 mM Tris-HCl (pH 6.8), 2.5% SDS, 10% glycerol, 5% β-mercaptoethanol, and 0.002% bromophenol blue. Lysates were incubated at 99°C for 5 min to ensure complete protein denaturation. Whole-cell lysates were then resolved by SDS–PAGE using 5–12% polyacrylamide gels and transferred onto PVDF membranes. Immunoblotting was performed using the indicated primary antibodies, and protein bands were detected using an enhanced chemiluminescence system (SuperSignal^™^ West Pico PLUS, Thermo Scientific, 34580). Densitometric analysis of immunoblots was carried out using ImageJ software.

### Aggresome Detection

Aggresome formation was assessed using the PROTEOSTAT Aggresome Detection Kit (ENZ-51035) according to the manufacturer’s instructions. After 72 hrs of treatment, cells were incubated with the detection reagent (1:5000 dilution) for 30 minutes at 37°C in the dark. For flow cytometry, cells were trypsinized, washed, and analyzed using the FL3 channel of a flow Cytometer (10,000 events/sample). For fluorescence microscopy, cells were fixed with 4% paraformaldehyde, counterstained with DAPI, and imaged using a fluorescence microscope. Aggresome-positive cells displayed a perinuclear red signal.

### Establishment of Autophagic Flux Reporter (AFR) Cell Lines

To establish autophagic flux reporter (AFR) cell lines, ovarian cancer cells (OVCAR8 and OVCA420) were transduced with a retroviral vector encoding the pBabe-mCherry-eGFP-LC3 construct (Addgene plasmid #22418; contributed by Jayanta Debnath). Eighteen hours after transduction, the viral supernatant was replaced with fresh complete medium. OVCAR8 cells were routinely grown in RPMI-1640 (Corning, 15–040-CV) supplemented with 2 mM L-glutamine and 10% fetal bovine serum (FBS). OVCA420 cells were routinely grown in MEM (Minimum Essential Medium) (Corning, 10–009-CVR) supplemented with 2 mM L- glutamine, 100mM Sodium Pyruvate, Non-essential amino acids, Vitamin, and 10% FBS. Expression of both eGFP and mCherry was verified by fluorescence microscopy at 48 hrs post-infection. Cells were then subjected to selection in complete medium containing puromycin (0.5 μg/ml) for 48–72 hrs. To optimize sensitivity and reproducibility, transduced cells were subjected to flow cytometric sorting and evaluated for their capacity to report autophagic flux as described previously (30). The resulting AFR cell lines (OVCAR8-AFR, OVCA420-AFR) were maintained in the previously described culture medium supplemented with puromycin (0.5 μg/ml) to preserve selective pressure.

### siRNA Transfection Protocol

Targeted gene silencing was achieved using siGENOME siRNA pools specific to SIK2 (M-004778-03-0005), SIK3 (M-004779-03-0005), ATF4 (M-005125-02-0005), CCPG1 (M-013998-01-0005) and CHOP (M-004819-03-0005), along with a non-targeting control pool (D-001210-01-05) (Horizon Discovery). siRNAs were reconstituted in 1X siRNA Buffer (Horizon Discovery, B-002000-UB-100) to a stock concentration of 20 μM, following the manufacturer’s guidelines. For transfection, 25 nM siRNA and 0.2% DharmaFECT 4 transfection reagent (Horizon Discovery, T-2001-02) were each diluted separately in Opti-MEM (Thermo Fisher Scientific, 31985070), then combined and incubated at room temperature for 30 minutes to allow complex formation. Cells were seeded directly onto the siRNA–DharmaFECT complexes. Seventy-two hrs of post-transfection, cells were harvested for gene and protein expression analysis or prepared for aggresome detection assays.

### Cell Viability Assays

Cell viability was assessed using the CellTiter-Glo Luminescent Cell Viability Assay (Promega, G9241). Between 2,500 and 3,000 cells per well were seeded into 96-well plates and treated 24 hours later with GRN-300, CQ, or their combination across a range of concentrations in serial dilutions. Following 72 hrs of treatment, the culture medium was removed, and each well received 60 μL of fresh medium mixed with 30 μL of CellTiter-Glo reagent. After 15 min of shaking, luminescence was recorded using a Synergy2 microplate reader (BioTek). Dose-response curves were generated, and half-maximal inhibitory concentrations (IC_50_) were determined using nonlinear regression analysis with normalized response and variable slope models in GraphPad Prism 9. Drug interactions were assessed using a fixed-ratio combination approach, and synergy was quantified using CalcuSyn 2.0 software (BIOSOFT) to calculate the combination index (CI). A CI < 1 indicated synergy, CI = 1 indicated an additive effect, and CI > 1 indicated antagonism.

### Clonogenic Assay

To assess the long-term proliferative capacity of cancer cells following treatment, clonogenic assays were performed in 12-well plates. Single-cell suspensions were seeded in triplicate at a density of 500–600 cells per well, adjusted based on the doubling time of each cell line. Twenty-four hours post-seeding, cells were exposed to one or two agents at various concentrations.

At the end of the incubation period, colonies were fixed with 4% paraformaldehyde for 15 min, washed with PBS, and stained with 0.5% crystal violet for 30 min at room temperature. After removing excess stain with distilled water, the plates were air-dried. Colony areas were counted using ImageJ software.

### Caspase-Glo 3/7 Assay

The mechanisms of cell death induced by GRN-300, chloroquine (CQ), or their combination were evaluated using the Caspase-Glo^®^ 3/7 Assay (Promega, G8093) according to the manufacturer’s instructions. Briefly, cells were seeded into white, opaque 96-well plates at a density of 3 × 10^3^ cells per well in growth medium and allowed to adhere overnight. The following day, the medium was replaced with fresh medium containing GRN-300, CQ, or their combination, and cells were incubated for an additional 72 hours. Caspase-Glo^®^ 3/7 reagent was then added to each well, and plates were shaken at 300 rpm for 30 seconds. Plates were incubated at room temperature for 60 minutes protected from light, and luminescence was measured using a BioTek multi-mode plate reader.

### Annexin V Apoptosis Assay

Apoptotic cell death induced by GRN-300, CQ, or their combination was quantified in ovarian cancer cell lines using the FITC Annexin V/Dead Cell Apoptosis Kit I (Thermo Fisher Scientific, V13242), following the manufacturer's protocol. Briefly, after the specified treatments, cells were harvested and washed once with 1× PBS. Cells were then resuspended in 1× binding buffer supplemented with 5 μL FITC-conjugated annexin V and 100 μg/mL propidium iodide (PI). Following a 15-min incubation at room temperature in the dark, cells were centrifuged at 1,000 × g for 3 minutes and resuspended in 200 μL of fresh 1× binding buffer. Flow cytometric analysis was performed using a Gallios Analyzer (Beckman Coulter), with a minimum of 10,000 events collected per sample at the MD Anderson Flow Cytometry Core lab. The percentage of early and late apoptotic cells was determined based on annexin V and PI staining profiles by using FlowJo V10 software.

### RNA Extraction and Quantitative RT-PCR Analysis

Ovarian cancer cells were treated with or without GRN-300 for 72 hrs, after which total RNA was isolated using TRIzol reagent (Thermo Fisher Scientific, 15596026) followed by purification with the RNeasy Mini Kit (Qiagen, 217004), in accordance with the manufacturer's protocols. First-strand cDNA synthesis was performed using 2 μg of total RNA with the SuperScript II First-Strand Synthesis System (Invitrogen, 11904–018), following the manufacturer’s instructions. Quantitative real-time PCR (RT-qPCR) was performed on a CFX Connect Real-Time PCR Detection System (Bio-Rad) in 20 μL reactions containing 10 μL of 2× SsoAdvanced Universal SYBR Green Supermix (Bio-Rad), gene-specific primers, and 5 ng of cDNA template. The forward primer (5′-AATCAGCTTCTGGTCCATCG-3′) and reverse primer (5′-TTCTTTTCCCTCCGTGCTC-3′) were used for the p62 gene. The forward primer (GTTACCCTTGAGCCACCTAAG3’) and reverse primer (5’ AGAGGAAGAGCCCATGTTAAAG 3’) were used for the CCPG1 gene. Thermocycling conditions consisted of an initial denaturation step at 95°C for 2 min, followed by 40 amplification cycles of 95°C for 5 seconds and 60°C for 30 sec. Relative mRNA expression levels were calculated using the 2^–ΔΔCt method, with GAPDH serving as the internal reference gene.

### ER-lysosome co-localization analysis

ER-lysosome co-localization was assessed using live-cell fluorescent labeling with ER-Tracker and LysoTracker dyes. Cells were seeded onto glass-bottom chamber slides and allowed them to adhere overnight. Following the indicated treatments, cells were incubated with ER-Tracker^™^ Green (Thermo Fisher Scientific, E34251; 1 μM) and LysoTracker^™^ Red DND-99 (Thermo Fisher Scientific, L7528; 50 nM) in pre-warmed complete culture medium for 30 min at 37°C. After staining, cells were gently washed once with pre-warmed PBS and maintained in phenol red–free medium for imaging. Nuclei were counterstained with DAPI (1 μg/mL) for 5 min at room temperature, followed by a brief PBS wash. Live-cell fluorescence images were acquired using an epifluorescence microscope equipped with FITC, Texas Red, and DAPI filter sets. Images were captured sequentially to minimize spectral bleed-through, and all exposure times, gain, and illumination settings were kept constant across conditions. Colocalization between ER and lysosomes was quantified using the Coloc2 plugin in Fiji (ImageJ). Background-subtracted images were analyzed to calculate Manders’ overlap coefficients (M1 and M2), representing the fraction of ER signal overlapping with lysosomes and vice versa. Thresholds were automatically determined using the Costes method to minimize background influence. Statistical significance among the four groups was determined using one-way ANOVA followed by Tukey’s multiple comparison test (GraphPad Prism).

### Ovarian cancer xenograft models using SKOv3, OC316, and OVCAR8 cell lines

A total of 120 female athymic (nu/nu) mice were used to establish xenografts: 5 × 10^6^ SKOv3 cells were injected subcutaneously (s.c.), 3 × 10^6^ OC316 cells s.c., and 3.5 × 10^6^ OVCAR8 cells intraperitoneally (i.p.). Seven days post-inoculation, animals were randomized into four treatment groups (n = 10 per group): (a) vehicle control, (b) GRN-300 (50 mg/kg, orally, five times per week), (c) CQ (40 mg/kg, i.p., five times per week), and (d) combination treatment with GRN-300 and CQ. Treatments were administered for six weeks in both SKOv3 and OC316 models, and the same duration was followed for OVCAR8. The mice were euthanized when average tumor volume in the vehicle-treatment arm reached 1000–1500 mm^3^. Euthanasia was carried out using an euthanex system for the delivery of CO2 followed by cervical dislocation. In the SKOv3 and OC316 models, tumor dimensions were recorded weekly using a digital caliper, and volumes were calculated using the formula: volume (mm^3^) = 0.5 × a × b^2^, where a and b represent the longest and shortest tumor diameters, respectively. Mice were monitored until tumors reached an ethical endpoint of 1500 mm^3^. In the OVCAR8 model, tumors were excised and weighed post-mortem immediately.

### Immunohistochemistry

Immunohistochemical staining was performed at the MD Anderson Pathology Core lab. Briefly, formalin-fixed, paraffin-embedded mouse xenograft tissues were sectioned and processed on a Leica Bond RX automated staining platform. Automated deparaffinization and rehydration were performed using BOND Dewax Solution (Leica, AR9222), graded alcohols, and BOND Wash Solution (Leica, AR9590). Antigen retrieval was carried out using BOND^™^ Epitope Retrieval ER1 Solution (Leica, AR9961) at 100°C for 20 min. Endogenous peroxidase activity was quenched using Refine Detection Kit Peroxide Block (Leica, DS9800) for 5 min, followed by blocking with 5% goat serum (Cell Signaling Technology, 5425) for 30 min at room temperature.

Sections were incubated with primary antibodies at room temperature as follows: Ki-67 (Abcam, 16667; 1:800) for 45 min and cleaved caspase-3 (Biocare, CP229B; 1:100) for 60 min. Signal detection was performed using the Refine Detection Kit Polymer (Leica, DS9800) for 8 min, followed by chromogenic development with Refine Detection Kit Mixed DAB (Leica, DS9800). All washing steps were performed automatically using BOND Wash Solution. Slides were counterstained with hematoxylin to visualize nuclei. Stained sections were examined using a light microscope, and representative images were acquired using a digital camera. Positive control tissues and negative control sections in which the primary antibody was omitted were included to confirm staining specificity and assess nonspecific background.

### Statistical analysis

Statistical analyses were performed using GraphPad Prism software. Data are presented as mean ± SD. Statistical significance between groups was determined using unpaired Student’s *t*-test or one way/two-way analysis of variance (ANOVA), as appropriate. All experiments were independently repeated at least three times, with technical triplicates measured for each condition.

## Results

### Inhibition of SIK2/3 disrupts proteostasis and activates the unfolded protein response.

**Figure 1:**
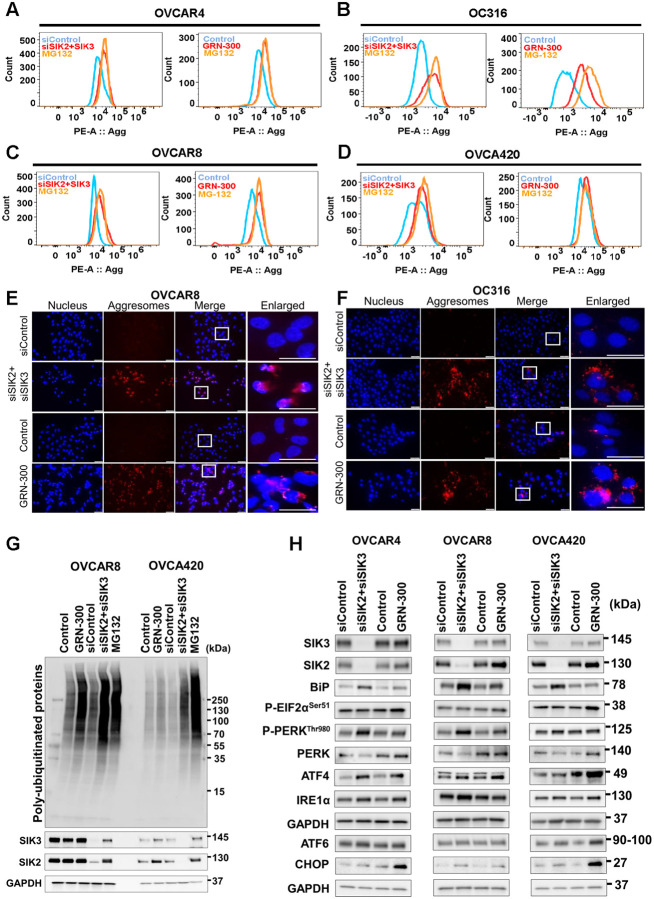
SIK2/3 inhibition promotes protein aggregation and triggers unfolded protein response (UPR) signaling. **(A-D)** Accumulation of aggregated proteins was assessed by flow cytometry following pharmacologic or genetic inhibition of SIK2/3. Ovarian cancer cells were treated with the SIK2/3 inhibitor GRN-300 (OVCAR4 and OVCAR8: 2 μM, OVCA420 and OC316: 4 μM) for 72 hrs or transfected with siRNAs targeting SIK2/3 (35 nM) for 72 hrs. **(E-F)**Protein aggregation was assessed by fluorescence microscopy (Scale bar: 50 μm). **(G)** Immunoblot analysis of whole-cell lysates treated as in (A-F) was performed, and analyzed for polyubiquitinated proteins following SIK2/3 inhibition. **(H)** Activation of the UPR signaling upon pharmacologic or genetic inhibition of SIK2/3. Cells treated with GRN-300 or subjected to combined SIK2/3 knockdown were analyzed by immunoblotting for the indicated UPR signaling components. Densitometric quantification was performed using ImageJ and is shown in Figure S1.

To maintain proteostasis under stress conditions, cancer cells rely on coordinated protein quality control mechanisms (8,31). We therefore investigated whether inhibition of SIK2/3 disrupts cellular proteostasis. We first examined whether suppression of SIK2/3 affects protein quality control by assessing intracellular protein aggregation following pharmacologic inhibition or genetic depletion in OVCAR4, OC316, OVCAR8, and OVCA420 ovarian cancer cells. Flow cytometric analysis using the PROTEOSTAT Aggresome Detection Kit revealed a clear rightward shift in fluorescence intensity in cells treated with the SIK2/3 inhibitor GRN-300 or subjected to combined siRNA-mediated knockdown of SIK2/3, comparable to that observed in cells treated with the proteasome inhibitor MG132 used as a positive control (Fig. 1A–D). Consistently, fluorescence microscopy demonstrated increased accumulation of PROTEOSTAT-positive aggregates in GRN-300-treated or SIK2/3-depleted cells, similar in magnitude to MG132-treated cells (Fig. 1E, F) in OVCAR8 and OC316 cells. In parallel, western blot analysis showed a pronounced increase in polyubiquitinated proteins under both conditions in OVCAR8 and OVCA420 cells, consistent with impaired protein clearance and induction of proteotoxic stress (Fig. 1G). Given that disruption of proteostasis is a well-established trigger of ER stress, we next examined activation of the UPR. Treatment with the SIK2/3 inhibitor GRN-300 or combined depletion of SIK2/3 resulted in activation of the PERK pathway, as evidenced by increased PERK phosphorylation and induction of the downstream effectors ATF4 and CHOP (Fig. 1H and S1) in OVCAR4, OVCAR8 and OVCA420 cells. Phosphorylation of eIF2α was modest and variable across cell lines, consistent with the transient nature of this modification. In parallel, the IRE1 branch of the UPR was also activated, whereas ATF6 protein levels were not significantly altered (Fig. 1H and S1). Importantly, expression of the ER chaperone BiP (GRP78), a canonical marker of ER stress and UPR activation, was increased following GRN-300 treatment or combined SIK2/3 depletion, further supporting induction of ER stress. These findings indicate that inhibition of SIK2/3 induces proteostasis imbalance and engages PERK- and IRE1-dependent ER stress signaling.

### Inhibition of SIK2/3 induces autophagy and enhances ER stress–associated autophagic flux.

**Figure 2:**
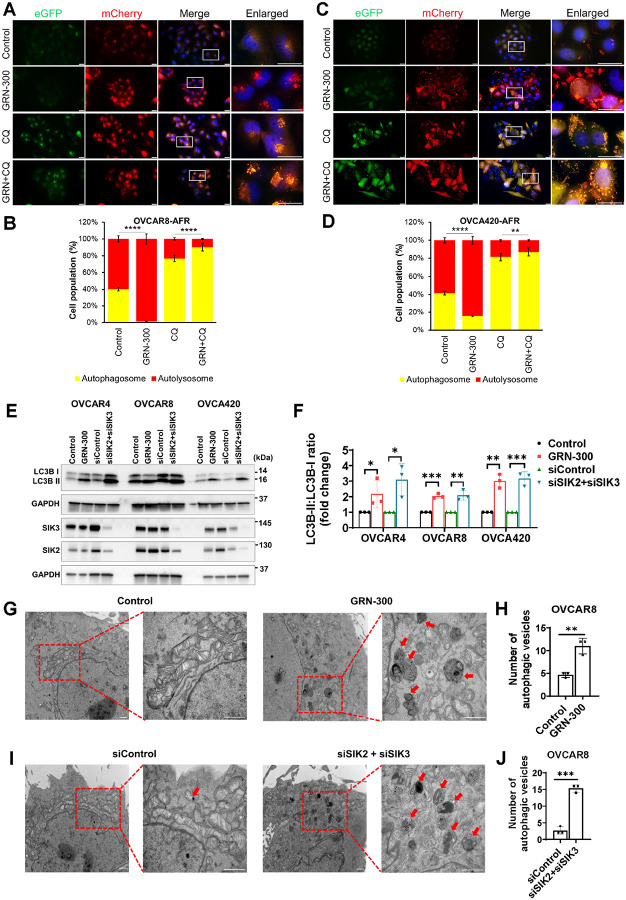
SIK2/3 inhibition induces autophagy and enhances ER stress-associated autophagic flux. **(A-D)** Autophagic flux was evaluated in autophagic flux reporter (AFR) OVCAR8-AFR and OVCA420-AFR cells expressing tandem mCherry-eGFP-LC3B following pharmacologic or genetic SIK2/3 inhibition. Cells were treated with GRN-300 (2 μM, 24–48 hrs) or transfected with siSIK2/3 (25 nM, 72 hrs). Statistical testing was performed from three independent high-power fields per condition using two-sided unpaired t-tests comparing the autolysosome (mCherry-LC3) signal between control vs GRN-300 and CQ vs CQ+GRN-300; **p < 0.01, ****p < 0.0001 (scale bar, 50 μm). Error bars represent SD. Data represents three independent experiments. **(E-F)**Western blot analysis of LC3B lipidation following SIK2/3 inhibition. OVCAR4, OVCAR8, and OVCA420 cells were treated with GRN-300 or subjected to combined SIK2/3 knockdown as described in (A-D). LC3B-I and LC3B-II levels were analyzed by immunoblotting. The LC3B-II/LC3B-I ratio was quantified using ImageJ, normalized to loading controls, and expressed relative to control cells. Data are presented as mean ± SD. Statistical significance was determined using an unpaired two-sided Student’s t-test; *p < 0.05; **p < 0.01; ***p < 0.001. **(G-J)** Ultrastructural analysis of autophagosome formation by TEM. OVCAR8 cells were treated with GRN-300 (2 μM, 72 hrs) or transfected with siSIK2/3 (25 nM, 72 hrs). Double-membraned autophagic vesicles were quantified from the indicated number of cells/fields (scale bar, 1 μm). Significance was determined by unpaired two-sided Student’s t-test.; **p < 0.01, ***p < 0.001. Error bars represent SD.

Accumulation of misfolded proteins and activation of the UPR are well-established triggers of autophagy, which functions as an adaptive mechanism to restore proteostasis under ER stress conditions (32). Given that inhibition of SIK2/3 induces proteotoxic stress and robust activation of PERK- and IRE1-dependent UPR signaling, we next investigated whether suppression of SIK2/3 engages autophagy as a cytoprotective response in ovarian cancer cells. To directly assess autophagic flux, we employed tandem GFP-mCherry-LC3 reporter cell lines (OVCAR8-AFR and OVCA420-AFR), which distinguish early autophagosomes from mature autolysosomes based on the differential stability of the fluorophores in acidic lysosomal compartments. Under basal conditions, LC3-positive puncta were predominantly GFP- and mCherry-positive, consistent with early-stage autophagosomes. In contrast, treatment with the SIK2/3 inhibitor GRN-300 or combined siRNA-mediated depletion of SIK2/3 significantly increased the proportion of mCherry-only puncta in both cell lines, indicating efficient autophagosome-lysosome fusion and enhanced lysosomal degradation (Fig. 2A–D). These results demonstrate that SIK2/3 inhibition induces a functional increase in autophagic flux rather than impairing autophagosome turnover. Consistent with these findings, immunoblot analysis revealed a marked increase in LC3B lipidation following either pharmacologic or genetic inhibition of SIK2/3, as evidenced by elevated LC3B-II levels and an increased LC3B-II:LC3B-I ratio in OVCAR4, OVCAR8, and OVCA420 cells (Fig. 2E, F), supporting enhanced autophagosome formation. Ultrastructural analysis by transmission electron microscopy provided direct morphological evidence of autophagy induction. GRN-300–treated and SIK2/3-depleted OVCAR8 cells exhibited a substantial increase in the number of double-membraned autophagic vesicles compared with control cells (Fig. 2G–J and S2), further confirming robust activation of autophagy at the ultrastructural level. Collectively, these findings demonstrate that inhibition of SIK2/3 triggers ER stress-associated autophagy and promotes a functional increase in autophagic flux, consistent with an adaptive proteostatic response in ovarian cancer cells.

### Inhibition of SIK2/3 activates ATF4-dependent CCPG1-mediated ER-phagy

**Figure 3:**
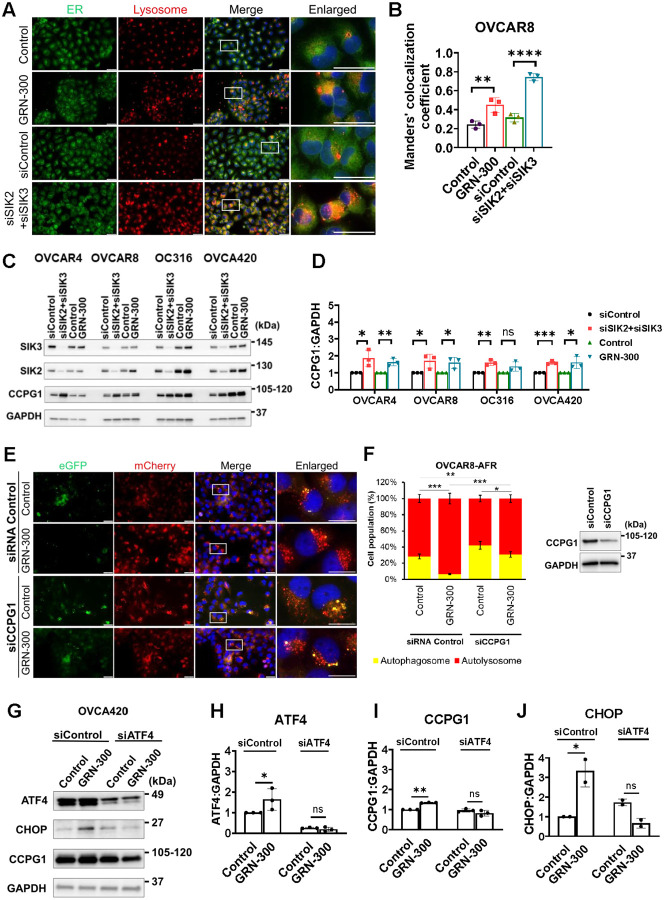
Inhibition of SIK2/3 activates ATF4-dependent CCPG1-mediated ER-phagy. **(A-B)** ER-lysosome colocalization following SIK2/3 inhibition. OVCAR8 cells were treated with GRN-300 (2 μM, 72 hrs) or transfected with siSIK2/3 (25 nM, 72 hrs). Colocalization was quantified by Manders’ coefficient (M1) using Coloc2 (Fiji). Data represent mean ± SD from three independent experiments. Significance was determined by one-way ANOVA with Tukey’s post hoc test (**p < 0.01; ****p < 0.0001) (scale bar, 50 μm). **(C-D)** Induction of the ER-phagy receptor CCPG1 following SIK2/3 inhibition. Cells were treated as in (A-B), and CCPG1 protein levels were analyzed by immunoblotting. Band intensities were quantified using ImageJ, normalized to loading controls, and expressed relative to control cells. Data represents three independent experiments. Statistical significance was determined by unpaired two-sided Student’s t-test (ns, p > 0.05; *p<0.05; **p < 0.01). Error bars indicate SD. **(E, F)** Requirement of CCPG1 for GRN-300–induced autophagic flux. OVCAR8 cells expressing tandem mCherry-eGFP-LC3B were transfected with control or CCPG1 siRNAs (25 nM, 24 hrs) and treated with GRN-300 (2 μM, 48 hrs). Statistical testing was performed from three independent high-power fields per condition using one-way ANOVA comparing the autolysosome (mCherry-LC3) signal between control and experimental groups; *p < 0.05, ***p < 0.001, ****p < 0.0001 (scale bar, 20 μm). Error bars represent SD. Data represents three independent experiments. **(G–J)** Regulation of CCPG1 expression by the PERK–ATF4 signaling axis. Cells were transfected with control or ATF4 siRNAs (25 nM, 24 hrs) and treated with GRN-300 for 48 h. CCPG1 and ATF4 protein levels were analyzed by immunoblotting. Band intensities were quantified using ImageJ, normalized to loading controls, and expressed relative to control conditions. Data represents three independent experiments. Statistical significance was determined by unpaired two-sided Student’s t-test (ns, p > 0.05; *p < 0.05; ****p < 0.0001). Error bars indicate SD.

Because unresolved ER stress can engage selective autophagy programs to eliminate damaged ER membranes, we next determined whether autophagy induced by SIK2/3 inhibition reflects activation of ER-phagy. Treatment with the SIK2/3 inhibitor GRN-300 or combined siRNA-mediated depletion of SIK2/3 significantly increased ER-lysosome colocalization in OVCAR8 cells, as assessed using ER- and lysosome-specific fluorescent trackers, indicating enhanced delivery of ER membranes to the lysosomal compartment (Fig. 3A, B).

Consistent with activation of an ER-phagy program, expression of cell cycle progression gene 1 (CCPG1), an ER stress-inducible ER-phagy receptor, was robustly upregulated at both the protein level in OVCAR4, OVCAR8, OC316, and OVCA420 cells (Fig. 3C) and at the mRNA level in OVCAR8 and OC316 cells (**Figure S3A**) following GRN-300 treatment or SIK2/3 knockdown. These findings suggested that SIK2/3 inhibition engages a CCPG1-associated ER stress response.

To determine whether CCPG1 is functionally required for SIK2/3 inhibition-induced autophagy, we assessed autophagic flux in mCherry-LC3 reporter (AFR) cell lines following CCPG1 depletion. In both OVCAR8-AFR and OVCA420-AFR cells, siRNA-mediated knockdown of CCPG1 markedly attenuated GRN-300-induced autophagic flux, as reflected by a significant reduction in the proportion of mCherry-only puncta compared with siRNA controls (Fig. 3E, F and S3B). Importantly, CCPG1 depletion substantially blunted the increase in flux observed after GRN-300 treatment, demonstrating that CCPG1 is required for the enhanced autophagic response triggered by SIK2/3 inhibition. Thus, CCPG1 functions as a critical effector of ER-phagy downstream of SIK2/3 inhibition rather than serving merely as a stress-induced marker.

To determine whether this response also reflects generalized bulk autophagy, we next evaluated the role of p62/SQSTM1. Although p62 expression was similarly increased at both protein and mRNA levels following GRN-300 treatment or SIK2/3 knockdown (**Figure S4A, B**), siRNA-mediated depletion of p62 failed to suppress GRN-300-induced autophagic flux in OVCAR8-AFR cells (**Figure S4C**). These findings indicate that, despite induction of multiple autophagy-associated proteins, the enhanced flux elicited by SIK2/3 inhibition is selectively dependent on CCPG1 and is unlikely to be mediated by p62-driven bulk autophagy. Together, these results establish CCPG1-dependent ER-phagy as the dominant autophagic pathway engaged upon SIK2/3 inhibition.

We next sought to define the upstream signaling mechanism governing CCPG1 induction. Given the robust activation of the PERK pathway following SIK2/3 inhibition, we focused on the PERK-ATF4 axis of the unfolded protein response. Genetic suppression of ATF4 abolished GRN-300-induced upregulation of CCPG1, as well as the canonical ATF4 target CHOP, at the protein level in OVCAR8 and OVCA420 cells (Fig. 3G–J and S4D). These results demonstrate that ATF4 is necessary for CCPG1 induction under conditions of SIK2/3 inhibition. Collectively, our data define a PERK-ATF4-CCPG1 signaling axis that mechanistically links SIK2/3 inhibition–induced ER stress to activation of adaptive ER-phagy in ovarian cancer cells.

### Autophagy inhibition potentiates the antitumor activity of GRN-300

**Figure 4:**
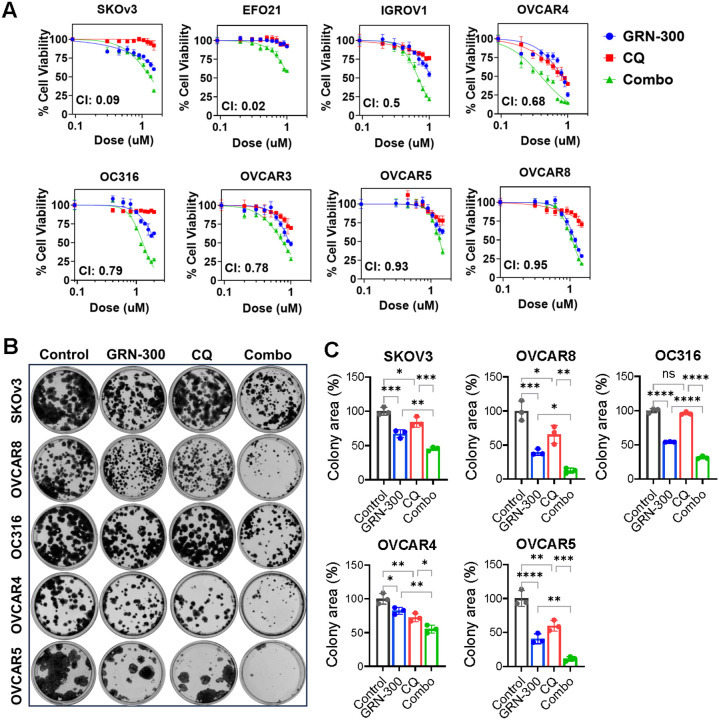
Autophagy inhibition potentiates the antitumor activity of GRN-300. **(A)** Cell viability following combined inhibition of SIK2/3 and CQ. Ovarian cancer cells were treated with the SIK2/3 inhibitor GRN-300 (2 μM), CQ (4–7 μM), or the combination for 72 hrs. Cell viability was measured using a luminescence-based Cell-Titer-Glo assay. Drug interactions were analyzed using the Chou–Talalay method, with a combination index (CI) < 0.9 considered indicative of synergy. Representative dose-response curves and corresponding CI values are shown. Experiments were performed at least three times with similar results. **(B, C)**Clonogenic survival following combined GRN-300 and CQ treatment. Ovarian cancer cells were treated with GRN-300 (2 μM), CQ (5 μM), or the combination as indicated and cultured for 10–14 days. Colonies were fixed, stained, and quantified. Representative images are shown, and the colony area was normalized to control. Experiments were repeated three times with similar results. Statistical analysis was performed using one-way ANOVA (ns, p > 0.05; *p < 0.05; **p < 0.01; ***p < 0.001; ***p < 0.0001). Error bars represent SD.

Given that inhibition of SIK2/3 induces robust ER stress and activates ER-phagy, we next examined whether autophagy functions as an adaptive, cytoprotective mechanism that enables ovarian cancer cells to tolerate proteotoxic stress. We therefore tested whether pharmacologic inhibition of autophagic flux sensitizes ovarian cancer cells to SIK2/3 inhibition. GRN-300 was combined with CQ, a lysosomal inhibitor that blocks autophagosome-lysosome fusion and impairs autophagic degradation. Co-treatment with GRN-300 and CQ resulted in a marked reduction in cell viability compared with either agent alone across multiple ovarian cancer cell lines, including SKOV3, EFO21, IGROV1, OVCAR4, OC316, OVCAR3, OVCA85, and OVCAR8 (Fig. 4A). Quantitative analysis using the Chou-Talalay method revealed strong synergistic interactions, with combination index (CI) values consistently below 0.9, indicating that autophagy inhibition unmasks a latent cytotoxic vulnerability induced by SIK2/3 inhibition. Consistent with these findings, clonogenic assays demonstrated that combined GRN-300 and CQ treatment significantly suppressed long-term colony formation relative to single-agent treatment (Fig. 4B–C and S5A). These results indicate that autophagy serves a protective role in response to SIK2/3 inhibition and that blockade of autophagic flux markedly enhances the antitumor efficacy of GRN-300 in vitro.

### Combined GRN-300 and CQ treatment suppresses tumor growth and prolongs survival in ovarian cancer xenograft models.

**Figure 5:**
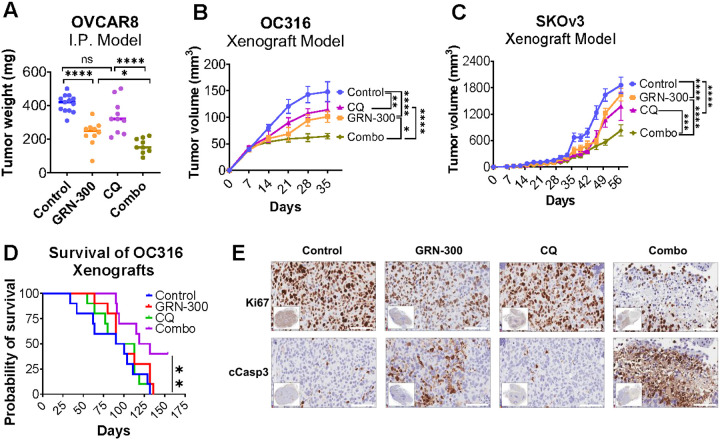
Autophagy inhibition enhances the anti-tumor efficacy of SIK2/3 inhibition in vivo. **(A-C)** Tumor growth and final tumor weight in ovarian cancer xenografts established in female athymic nu/nu mice treated with vehicle, single agents, or combination therapy (n = 10 per group). Tumor growth was monitored by caliper measurement and plotted as tumor weight at endpoint (A) or tumor volume (B, C), shown as mean ± SEM. Statistical analyses were performed using two-way ANOVA for tumor volume and one-way ANOVA for tumor weight (ns, p > 0.05; *p < 0.05; **p < 0.01; ***p < 0.001; ****p < 0.0001). The experiment was performed once. **(D)** Kaplan–Meier survival analysis of OC316 xenograft-bearing mice, using predefined ethical endpoints. Survival curves were generated using GraphPad Prism 10 and compared by the log-rank (Mantel-Cox) test (**p < 0.01). **(E)** Immunohistochemical staining of Ki67 and cleaved caspase-3 in OVCAR8 xenograft tumors. Representative images are shown (scale bar, 60 μm).

To determine whether the cytoprotective role of autophagy observed in vitro translates into therapeutic resistance in vivo, we evaluated the antitumor efficacy of GRN-300 in combination with autophagy inhibition in ovarian cancer xenograft models. Across three independent models (OVCAR8, OC316, and SKOV3), combined treatment with the SIK2/3 inhibitor GRN-300 and the lysosomal inhibitor CQ resulted in a marked suppression of tumor growth compared with either agent alone (Fig. 5A–C), demonstrating a robust and reproducible antitumor effect of the combination therapy. Consistent with tumor growth inhibition, survival analysis in the OC316 xenograft model revealed that mice receiving combined GRN-300 and CQ treatment exhibited significantly prolonged survival relative to monotherapy-treated cohorts (Fig. 5D). These findings corroborate our in vitro data and indicate that blockade of autophagic flux enhances the antitumor activity of SIK2/3 inhibition in vivo. To define the biological basis of this enhanced therapeutic response, we analyzed tumor tissues for markers of proliferation, apoptosis, and autophagy. Immunohistochemical analysis of OVCAR8 xenografts demonstrated that combined GRN-300 and CQ treatment markedly reduced expression of the proliferation marker Ki67 and significantly increased levels of cleaved caspase-3 (CASP3) compared with either single agent, indicating suppressed tumor cell proliferation and enhanced apoptotic cell death (Fig. 5E). Immunoblot analysis of tumor lysates corrected at end of study revealed that GRN-300 or CQ treatment alone increased levels of LC3B-I and LC3B-II, consistent with induction or accumulation of autophagic structures, whereas combination treatment reduced overall LC3B abundance (**Figure S5B**). This decrease likely reflects extensive tumor cell loss and reduced viable tumor mass following combination therapy rather than direct suppression of autophagosome formation as tumor. Collectively, these in vivo results demonstrate that autophagy inhibition potentiates GRN-300 induced ER stress and tumor cell death, resulting in suppressed tumor progression and extended survival.

### Autophagy inhibition converts GRN-300 induced ER stress into CHOP-dependent apoptotic cell death

**Figure 6:**
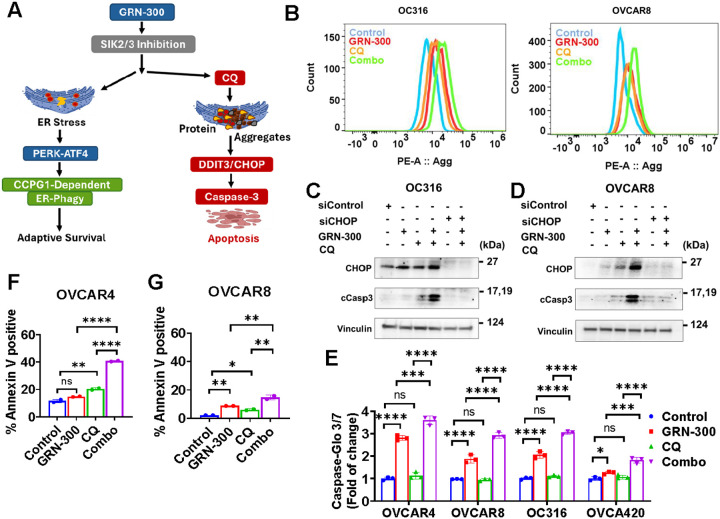
Autophagy inhibition converts GRN-300-induced ER stress into CHOP-dependent apoptotic cell death. **(A)** Proposed model of SIK2/3 inhibition induced ER stress, ER-phagy, and apoptosis in ovarian cancer. **(B)**Protein aggregation following combined GRN-300 and CQ treatment. OVCAR8 (2 μM GRN-300) and OC316 (4 μM GRN-300) cells were treated for 72 hrs, and protein aggregation was assessed by flow cytometry. Representative images are shown. Experiments were repeated three times with similar results. **(C-D)** Induction of CHOP and cleaved caspase-3 following combined inhibition of SIK2/3 and autophagy. Cells were treated with GRN-300, CQ, or the combination as indicated, and protein levels were analyzed by immunoblotting. **(E)** Quantification of caspase activation. Ovarian cancer cells were treated with GRN-300 (2 μM), CQ (7.5 μM), or the combination for 72 hrs and caspase activity was assessed by using Caspase-Glo 3/7. Experiments were performed three times independently with similar results. (**F-G**) Apoptosis analysis following combined GRN-300 and CQ treatment. Cells were treated as in (B), and apoptosis was assessed using Annexin V/PI staining followed by flow cytometry. Representative plots are shown, and the percentage of Annexin V–positive cells was quantified. Experiments were performed three times independently with similar results. Statistical analysis was conducted using one-way ANOVA (ns, p > 0.05; *p < 0.05; **p < 0.01; ***p < 0.001; ****p < 0.0001). Error bars represent SD.

Having established that SIK2/3 inhibition induces ER stress and activates CCPG1-dependent ER-phagy as an adaptive survival response, we next sought to determine how blockade of autophagic flux enhances the antitumor activity of the SIK2/3 inhibitor GRN-300. We propose a model in which SIK2/3 function as previously unrecognized regulators of ER stress adaptation in ovarian cancer cells (Fig. 6A). Inhibition of SIK2/3 disrupts proteostasis, leading to accumulation of misfolded proteins and activation of the unfolded protein response, with dominant engagement of the PERK-ATF4 signaling axis. ATF4-dependent induction of the ER-phagy receptor CCPG1 selectively activates ER-phagy, enabling clearance of damaged ER membranes and aggregated proteins and thereby restoring ER homeostasis and cell survival. This adaptive ER-phagy response acts as a critical survival checkpoint that restrains the cytotoxic consequences of SIK2/3 inhibition. Pharmacologic blockade of autophagic flux prevents resolution of ER stress, resulting in proteostasis collapse, sustained ATF4 signaling, and induction of CHOP-dependent apoptosis. Consistent with this model, co-treatment with GRN-300 and CQ, a lysosomal inhibitor, in OVCAR8 and OC316 cells resulted in a pronounced rightward shift in fluorescence intensity compared with either agent alone, as assessed by flow cytometric analysis of protein aggregates (Fig. 6B). These findings indicate a failure of proteostasis maintenance upon inhibition of autophagic degradation. Escalation of proteotoxic stress was accompanied by increased expression of CHOP and cleaved CASP3, as determined by immunoblot analysis, consistent with unresolved ER stress and activation of apoptotic signaling. Importantly, siRNA-mediated depletion of CHOP markedly attenuated GRN-300- and CQ-induced CASP3 cleavage in the same immunoblot analyses (Fig. 6C, D), establishing CHOP as a critical mediator linking unresolved ER stress to apoptosis under conditions of impaired autophagic flux. Induction of apoptosis by combined GRN-300 and CQ treatment was further validated using Caspase-Glo 3/7 assays in OVCAR4, OVCAR8, OC316, and OVCA420 cells (Fig. 6E), and by Annexin V/propidium iodide staining in OVCAR8 and OC316 cells (Fig. 6F, G). Combination treatment consistently resulted in increased caspase activation and a substantial rise in Annexin V- and propidium iodide-positive cells compared with monotherapy-treated cells (Fig. 6E–G and S6), confirming induction of apoptotic cell death. These in vitro findings are concordant with our in vivo observations, as immunohistochemical analysis of ovarian cancer xenograft tumors revealed increased cleaved CASP3 staining and a concomitant reduction in expression of the proliferation marker Ki67 following combined GRN-300 and CQ treatment (Fig. 5E). Together, these results demonstrate that autophagy functions as a key adaptive mechanism that restrains GRN-300 induced proteotoxic and ER stress. Pharmacologic inhibition of autophagic flux overwhelms this protective response, leading to proteostasis collapse, CHOP-dependent apoptotic cell death, and enhanced antitumor efficacy both in vitro and in vivo.

## Discussion

In this study, we identify GRN-300, a selective inhibitor of the salt-inducible kinases SIK2/3, as a previously unrecognized pharmacologic inducer of ER stress in ovarian cancer cells. Unlike canonical ER stressors such as tunicamycin or thapsigargin, which directly disrupt protein folding or calcium homeostasis within the ER lumen (33,34), SIK2/3 inhibition induces ER stress indirectly through perturbation of upstream kinase signaling. These findings position SIK2/3 as critical regulators of ER proteostasis and cellular stress adaptation, extending their established roles in metabolism, mitosis, and survival signaling into the regulation of protein quality control.

Pharmacologic or genetic inhibition of SIK2/3 triggered robust activation of the UPR, with prominent engagement of the PERK-ATF4 and IRE1 signaling branches. Activation of the PERK-ATF4 axis was accompanied by induction of ER stress-responsive genes, including CCPG1, an ER-resident autophagy receptor that mediates selective degradation of damaged ER membranes. These findings are consistent with prior work demonstrating that UPR signaling coordinates selective autophagy programs to alleviate ER stress and restore organelle homeostasis (35–37). Our data therefore uncovers a previously unappreciated mechanistic link between SIK signaling and ER stress induced ER-phagy, positioning SIK2/3 upstream of this adaptive quality control pathway.

A key finding of this study is that SIK2/3 inhibition activates autophagy in a productive manner, characterized by enhanced autophagic flux rather than impaired turnover. Tandem LC3 reporter assays and ultrastructural analyses demonstrated efficient autophagosome maturation and lysosomal degradation following SIK2/3 suppression. Mechanistically, the enhanced autophagic response appears to be preferentially mediated through ER-phagy. The ER-phagy receptor CCPG1 was robustly induced at both mRNA and protein levels following SIK2/3 inhibition, and its depletion markedly attenuated GRN-300 induced autophagic flux in mCherry-LC3 reporter cells, identifying CCPG1 as a key mediator of this response. In contrast, although p62 levels were modestly increased under the same conditions, this did not reflect impaired autophagic degradation. Both p62 mRNA and protein were transcriptionally induced following SIK2/3 siRNA or GRN-300 treatment, consistent with activation of stress-response pathways such as ATF4 and NRF2, which are known to upregulate p62 during cellular stress (38,39). Importantly, depletion of p62 did not significantly impair GRN-300 induced autophagic flux, indicating that p62 is unlikely functionally required for the autophagic response in this context. Together, these findings support a model in which SIK2/3 inhibition triggers a stress-adaptive autophagic program dominated by CCPG1-dependent ER-phagy rather than by canonical p62-mediated cargo recruitment.

Mechanistically, we further define a PERK-ATF4-CCPG1 signaling axis linking SIK2/3 inhibition induced ER stress to activation of adaptive ER-phagy. Genetic suppression of ATF4 abrogated CCPG1 induction, positioning ATF4 as an essential upstream regulator of this selective autophagy pathway. These findings provide a mechanistic framework in which SIK2/3 inhibition induces proteotoxic stress and PERK activation, leading to ATF4-dependent transcriptional upregulation of CCPG1 and engagement of ER-phagy to restore ER homeostasis.

Functionally, ER-phagy serves as a cytoprotective mechanism that constrains the cytotoxic consequences of GRN-300 induced proteotoxic stress. Although SIK2/3 inhibition activates ER stress signaling and selective autophagy, this response alone is insufficient to drive robust apoptosis. Instead, ovarian cancer cells depend on CCPG1-mediated ER-phagy to limit accumulation of misfolded proteins and damaged ER membranes, thereby preserving viability under stress conditions (18,37,40). This model is consistent with accumulating evidence that selective autophagy pathways enable tumor cells to tolerate metabolic stress, hypoxia, and therapeutic challenge (41,42). Our findings extend this concept by identifying SIK2/3 as upstream regulators of an adaptive ER-phagy program that can be therapeutically exploited.

Disruption of autophagic flux using CQ abrogated this adaptive response, resulting in excessive accumulation of protein aggregates, escalation of ER stress, and activation of DNA damage-inducible CHOP-dependent apoptosis. Mechanistically, CQ-mediated blockade of lysosomal degradation converted an otherwise adaptive ER stress response into a terminal apoptotic program by preventing resolution of proteotoxic stress. Genetic depletion of CHOP significantly attenuated apoptosis induced by combined GRN-300 and CQ treatment, establishing CHOP as a critical downstream effector linking unresolved ER stress to cell death. These findings reveal a synthetic lethal interaction between SIK2/3 inhibition and autophagy blockade, in which ER-phagy acts as a key survival checkpoint.

Importantly, this mechanism translated to robust antitumor activity in vivo. Combined GRN-300 and CQ treatment suppressed tumor growth and significantly prolonged survival in ovarian cancer xenograft models, accompanied by increased cleaved caspase-3 and reduced Ki67 expression in tumor tissues. These data indicate that autophagy inhibition amplifies GRN-300 induced ER stress beyond the threshold of cellular tolerance, resulting in durable tumor cell death rather than transient growth arrest. Given that CQ and hydroxy-CQ are clinically approved antimalarial agents, our findings support the potential feasibility of a translational strategy targeting ER stress vulnerabilities in ovarian cancer. Tumors with elevated basal ER stress or heightened dependence on selective autophagy, such as platinum-resistant disease may be particularly susceptible. Moreover, biomarkers identified in this study, including CCPG1, CHOP, and LC3B, may serve as pharmacodynamic indicators to guide patient selection and monitor therapeutic response.

Despite establishing a mechanistic connection between SIK2/3 inhibition, ER stress, ER-phagy, and apoptotic cell death, several important questions remain. First, the direct downstream substrates of SIK2/3 that link kinase inhibition to UPR activation and ER-phagy induction remain to be defined. Given that SIKs are members of the AMPK family and regulate transcriptional programs through CREB-CRTC signaling (43,44), future studies should determine whether SIK2/3 directly modulate ER stress sensors, autophagy-related transcription factors, or broader proteostasis networks. Second, while this study focuses on ovarian cancer, the extent to which SIK-dependent regulation of ER stress and ER-phagy operates across other malignancies remains to be explored. SIK2/3 are overexpressed in several tumor types, including pancreatic and triple-negative breast cancers, suggesting that this pathway represents a potentially exploitable vulnerability (45,46). Finally, although CQ provides a clinically relevant proof-of-concept for autophagy inhibition, its limited potency and pleiotropic effects underscore the need to evaluate next-generation lysosomal and autophagy inhibitors. Future studies should assess combination strategies with more selective agents, as well as with other proteostasis-targeting therapies such as proteasome inhibitors. Identification of predictive biomarkers such as basal ER stress signatures, ER-phagy capacity, or SIK2/3 expression will be critical for patient stratification and successful clinical translation.

In addition, beyond direct cytotoxicity, sustained ER stress has been implicated in promoting immunogenic cell death and shaping antitumor immune responses (47,48). Thus, combining SIK inhibition with autophagy blockade may also enhance sensitivity to immunotherapy, an avenue that warrants further investigation.

## Supplementary Material

This is a list of supplementary files associated with this preprint. Click to download.

- OriginalDataqPCRfigures.xlsx
- SupplementaryData05192026.docx
- WBuncropped.pdf

## Data Availability

All data generated or analyzed during this study are included in this published article and its supplementary information files. Additional information supporting the findings of this study is available from the corresponding author upon request.

## Significance

Salt-inducible kinases SIK2/3 enable ovarian cancer cells to survive ER stress by activating ATF4-dependent ER-phagy. Blocking autophagy converts this adaptive response into CHOP-dependent apoptosis, revealing a therapeutically exploitable stress vulnerability.
